# Development of endosome-related gene signature for the prediction of prognosis and therapeutic response in breast cancer

**DOI:** 10.1097/MD.0000000000041230

**Published:** 2025-01-10

**Authors:** Guowei Jiang, Ye Wang

**Affiliations:** a Department of Breast, Haining Maternity and Child Health Care Hospital, Haining, Zhejieng, China.

**Keywords:** breast cancer, endosomes, immune landscape, nomogram, therapeutic response

## Abstract

Endosomes play a pivotal role in cellular biology, orchestrating processes such as endocytosis, molecular trafficking, signal transduction, and recycling of cellular materials. This study aims to construct an endosome-related gene (ERG)-derived risk signature for breast cancer prognosis. Transcriptomic and clinical data were retrieved from The Cancer Genome Atlas and the University of California Santa Cruz databases to build and validate the model. A Lasso Cox regression model was employed for risk signature construction. The immune landscape was assessed using CIBERSORT and ESTIMATE algorithms, while drug sensitivity was evaluated via the pRRophetic algorithm. Gene set enrichment analysis and gene set variation analysis were applied to evaluate gene expression patterns. A nomogram was constructed and validated for predicting breast cancer outcomes. The expression of ERGs in breast cancer cells and tissues was further validated. Sixty-one ERGs associated with breast cancer prognosis were identified, with 23 selected for constructing the risk signature. This signature stratified breast cancer patients into high- and low-risk groups, where the low-risk group exhibited significantly better prognosis. Notably, younger patients tended to have lower risk scores compared to older ones. The low-risk group exhibited enhanced sensitivity to the majority of the drugs tested, accompanied by increased infiltration of T cells and M1 macrophages. Additionally, cell cycle pathways were suppressed in the low-risk group, whereas antigen binding functions were significantly activated. Ultimately, risk score and age were identified as independent prognostic factors for breast cancer, and these factors were incorporated into a nomogram that demonstrated excellent performance in prognosis assessment. Finally, external cohort validated the dysregulation of the risk score-associated ERGs in breast cancer cells and tissues. This study successfully established an ERG-derived breast cancer risk signature and nomogram, elucidating their potential value in prognosis prediction and evaluation of therapeutic response.

## 1. Introduction

Breast cancer, the most frequently diagnosed cancer and the leading cause of cancer-related deaths among women globally, represents a significant public health challenge. The disease’s heterogeneity and complex interactions within the tumor microenvironment (TME) contribute to its clinical complexity and therapeutic resistance.^[1]^ Despite remarkable advancements in early detection methods and therapeutic approaches,^[2]^ a substantial proportion of patients still progress to advanced stages, underscoring the crucial need for refined prognostic indicators and personalized treatment strategies.^[3]^

Endosomes, which are central to intracellular trafficking, have emerged as pivotal players in cancer biology. They play a pivotal role in modulating the TME, angiogenesis, and immune responses.^[4,5]^ These vesicular compartments not only mediate the degradation of extracellular materials but also facilitate intercellular communication via exosome secretion,^[6]^ with the latter implicated in cancer progression through oncogenic signaling, extracellular matrix remodeling, and immune evasion mechanisms.^[7,8]^ Given their reflection of cellular origin, exosome contents serve as a rich source of biomarkers for noninvasive diagnostics and hold promise for monitoring disease progression and therapeutic response.^[9–11]^

In this study, we aim to elucidate the role of endosome-related genes (ERG) in breast cancer prognosis. By leveraging high-throughput datasets and advanced bioinformatics tools, we seek to identify and validate an ERG signature that can predict patient outcomes and guide immunotherapy strategies, thereby addressing a crucial gap in breast cancer management.

## 2. Materials and methods

### 2.1. Data acquisition

Transcriptomic and clinical data were obtained from The Cancer Genome Atlas (TCGA, https://portal.gdc.cancer.gov/) for the TCGA-BRCA cohort. Genes with more than half of the samples showing zero expression were excluded, as were patients with a follow-up duration of <30 days, resulting in a total of 1095 cases available for signature development. Additionally, the Vijver (2002) cohort,^[12]^ consisting of 295 breast cancer cases from the University of California Santa Cruz database (https://xenabrowser.net/datapages/) was utilized for validation purpose. A total of 590 ERGs were sourced from the Molecular Signature Database (MSigDB, https://www.gsea-msigdb.org/gsea/msigdb/index.jsp) (Table S1, Supplemental Digital Content, http://links.lww.com/MD/O270). To further validate the expression of ERGs, gene expression data were collected from 66 breast cancer cell lines and 80 noncancerous cell lines sourced from the Cancer Cell Line Encyclopedia (https://sites.broadinstitute.org/ccle/datasets) database. Furthermore, transcriptomic data were downloaded from the Gene Expression Omnibus (https://www.ncbi.nlm.nih.gov/gds/) for the GSE14999 cohort, which includes 68 paired samples of breast cancer and adjacent normal tissue.

### 2.2. Differential expression analysis

Limma^[13]^ was employed to perform differential expression analysis on the TCGA-BRCA cohort (1113 tumor vs 113 normal samples), following voom transformation, with genes selected based on adjusted *P*-value < .05 and |log2(fold change)| > 1. Volcano plots were visualized using ggplot2.

### 2.3. Prognostic assessment

The association between ERGs and breast cancer prognosis was evaluated using univariate Cox proportional hazards regression models, implemented with the “survival” package. Kaplan–Meier (K–M) survival curves and log-rank tests were conducted to assess survival differences among patient groups, with results visualized using the ggsurvplot function from the “survminer” package.

### 2.4. Signature construction and evaluation

Lasso Cox regression was implemented using the “glmnet” package in R, with cross-validation via cv.glmnet. Genes with nonzero coefficients were selected to compute the risk score (riskscore = Σ(βi × exp_leveli)), and patients were classified into high- and low-risk groups based on the median score, followed by K–M analysis. The performance of the risk signature was assessed via receiver operating characteristic (ROC) analysis using the “survivalROC” package.

### 2.5. Enrichment analysis

Gene set enrichment analysis (GSEA) based on Gene Ontology and Kyoto Encyclopedia of Genes and Genomes pathway gene sets were conducted using “clusterProfiler” package,^[14]^ with results visualized via dotplots. Gene set variation analysis^[15]^ was applied to analyze ERG-related gene sets, and Wilcox test was performed to compare high and low-risk groups using the “stat_compare_means” function.

### 2.6. Analysis of therapeutic response

Drug sensitivity analysis was conducted using the pRRophetic package,^[16]^ which employed the predictedPtype function to assess sensitivity against a panel of 45 drugs. Correlation between ERG expression levels and drug sensitivity was evaluated using the corr.test function from the psych package, with visual representation facilitated by corrplot. The Tumor Immune Dysfunction and Exclusion (TIDE) online tool (http://tide.dfci.harvard.edu/), was utilized to gauge immune response to immunotherapy. Correlation between TIDE scores and risk scores (riskscore) was assessed by corr.test, and group differences were examined using the wilcox test.

### 2.7. Immune infiltration analysis

Tumor immune landscape assessment was carried out using the IOBR package,^[17]^ implementing the deconvo_tme function with both cibersort and estimate methodologies. Correlations between ERG profiles and immune cell infiltration were quantified using corr.test from the psych package, and Wilcox test was employed to discern differences in infiltration patterns between high- and low-risk groups.

### 2.8. Somatic mutation analysis

Analysis of somatic mutations was undertaken with the maftools package,^[18]^ featuring visualization through the oncoplot function.

### 2.9. Nomogram construction and evaluation

Univariate and multivariate Cox regression analyses were employed to identify prognostic factors for breast cancer, with the rms package facilitating nomogram development. Calibration curves were generated using the calibrate function to assess model accuracy. Decision curve analysis was performed utilizing the decision_curve function from the rmda package, visualized via plot_decision_curve. ROC curve analysis was further adopted to evaluate the predictive performance of the nomogram.

## 3. Results

### 3.1. Expression, mutation, and prognostic relevance of endosome-associated genes

**Figure 1.**
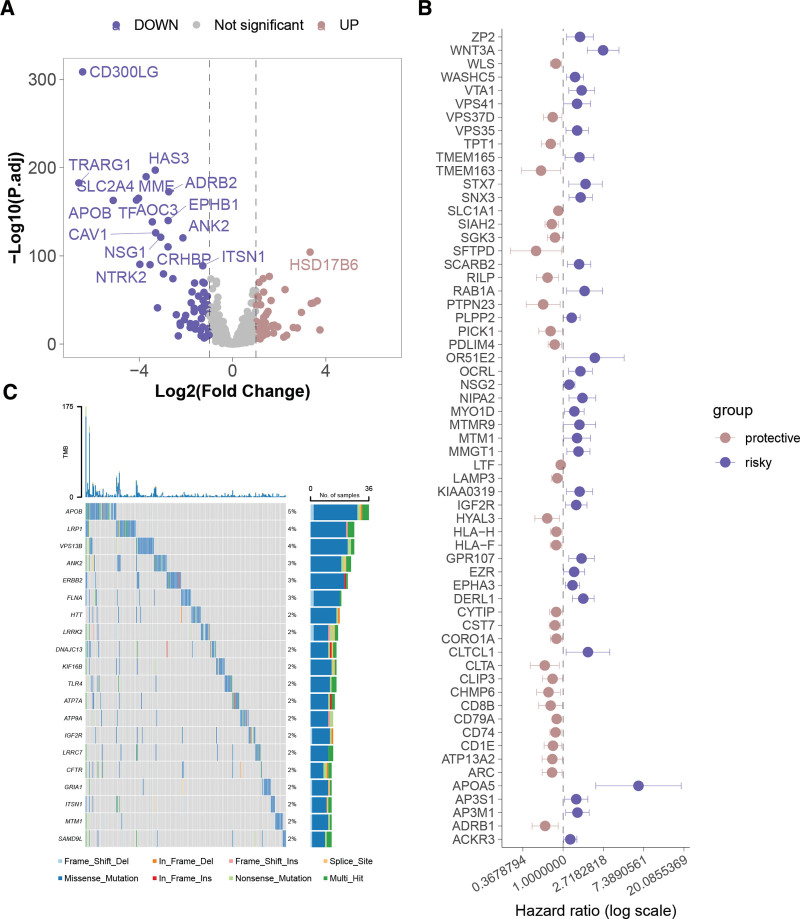
Expression, mutation, and prognostic associations of endosome-related genes in the TCGA-BRCA cohort. (A) Volcano plot illustrating differential expression of endosome-related genes (ERGs). (B) Hazard ratios for ERGs significantly associated with breast cancer prognosis. (C) Patterns of somatic mutations in ERGs. TCGA = The Cancer Genome Atlas.

Differential expression analysis identified 114 ERGs with altered expression in breast cancer, comprising 56 significantly overexpressed and 58 under expressed genes (Fig. 1A; Table S2, Supplemental Digital Content, http://links.lww.com/MD/O270). To assess the association between ERGs and breast cancer prognosis, univariate Cox regression analysis was performed, revealing 61 ERGs with significant prognostic relevance, wherein expression of 30 genes was found to be beneficial for prognosis, while 31 genes indicated an unfavorable prognosis (Fig. 1B; Table S3, Supplemental Digital Content, http://links.lww.com/MD/O270). The somatic mutation patterns of ERGs in breast cancer highlighted APOB, LRP1, and VPS13B as the top 3 frequently mutated genes (Fig. 1C).

### 3.2. Construction of a risk signature based on ERGs

**Figure 2.**
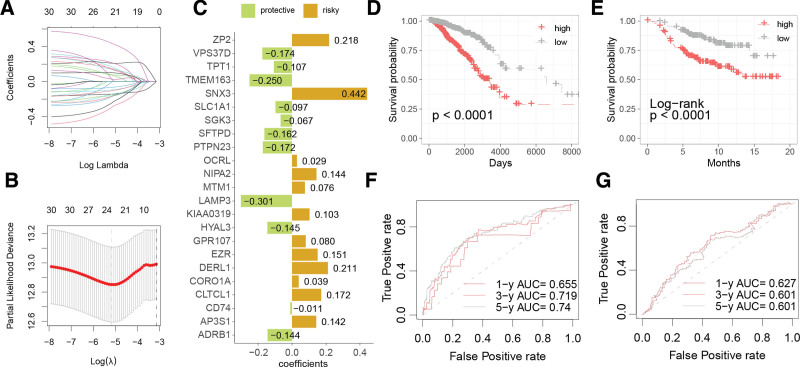
Construction and evaluation of a breast cancer risk signature derived from endosome-related genes (ERGs). (A and B) Lasso regression analysis for feature selection. (C) Coefficients of genes composing the risk score. Kaplan–Meier (K–M) survival analysis comparing the high- and low-risk groups in the TCGA-BRCA cohort (D) and Vijver (2002) cohort (E). Receiver operating characteristic (ROC) curves assessing the predictive performance of the risk score in the TCGA-BRCA cohort (F) and Vijver (2002) cohort (G). TCGA = The Cancer Genome Atlas.

Using Lasso Cox regression analysis, 23 ERGs were selected from the 61 prognostically relevant ones to construct a breast cancer risk signature (Fig. 2A and B). The coefficients of these genes contributing to the risk score are depicted in Figure 2C. Based on the median risk score, the TCGA-BRCA and Vijver (2002) cohorts were categorized into high- and low-risk groups, with subsequent K–M survival analysis demonstrating significantly worse outcomes for the high-risk group (Fig. 2D and E). The area under the curve (AUC) for predicting 1-, 3-, and 5-year overall survival by the risk score was 0.655, 0.719, and 0.74 in the TCGA-BRCA cohort, respectively, and 0.627, 0.601, and 0.601 in the Vijver (2002) cohort (Fig. 2F and G).

### 3.3. Characteristics of the ERG-derived risk signature

**Figure 3.**
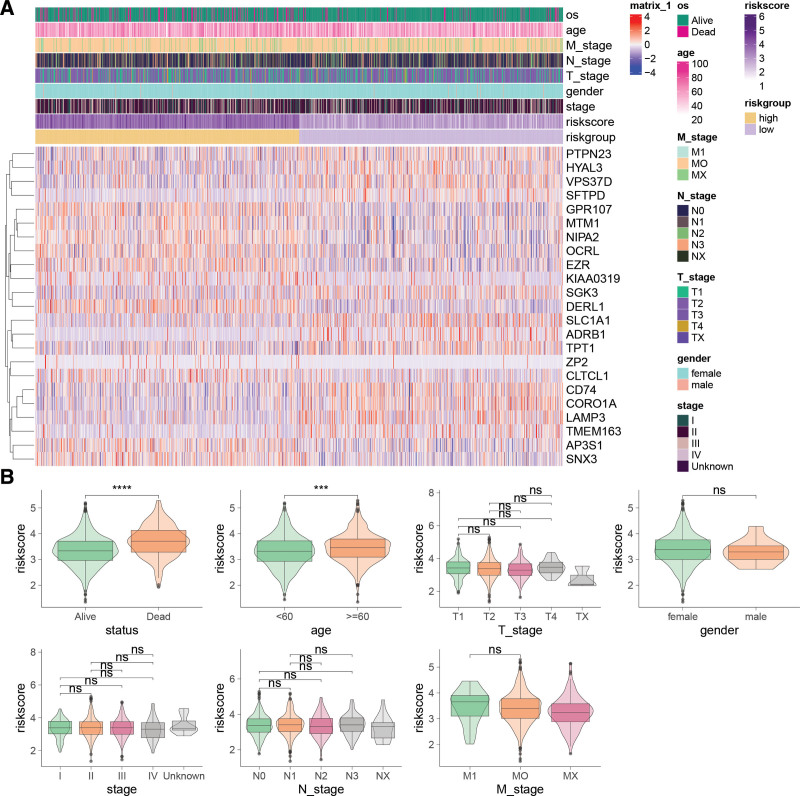
Association of the endosome-related genes (ERGs)-derived risk signature with clinical and pathological characteristics. (A) Heatmap depicting expression of genes forming the risk signature with annotations for clinical-pathological features. (B) Comparison of risk scores across different clinical-pathological subgroups. ns = not significant; ***P < .001; ****P < .0001.

An expression heatmap of the ERGs forming the risk signature (Fig. 3A) revealed correlations with overall survival and age. Notably, alive patients had significantly lower risk scores compared to deceased patients, and older patients (≥60 years) had higher risk scores than younger patients. However, no significant differences in risk scores were observed across genders or different stages (Fig. 3B).

### 3.4. Differential gene expression between high- and low-risk groups

**Figure 4.**
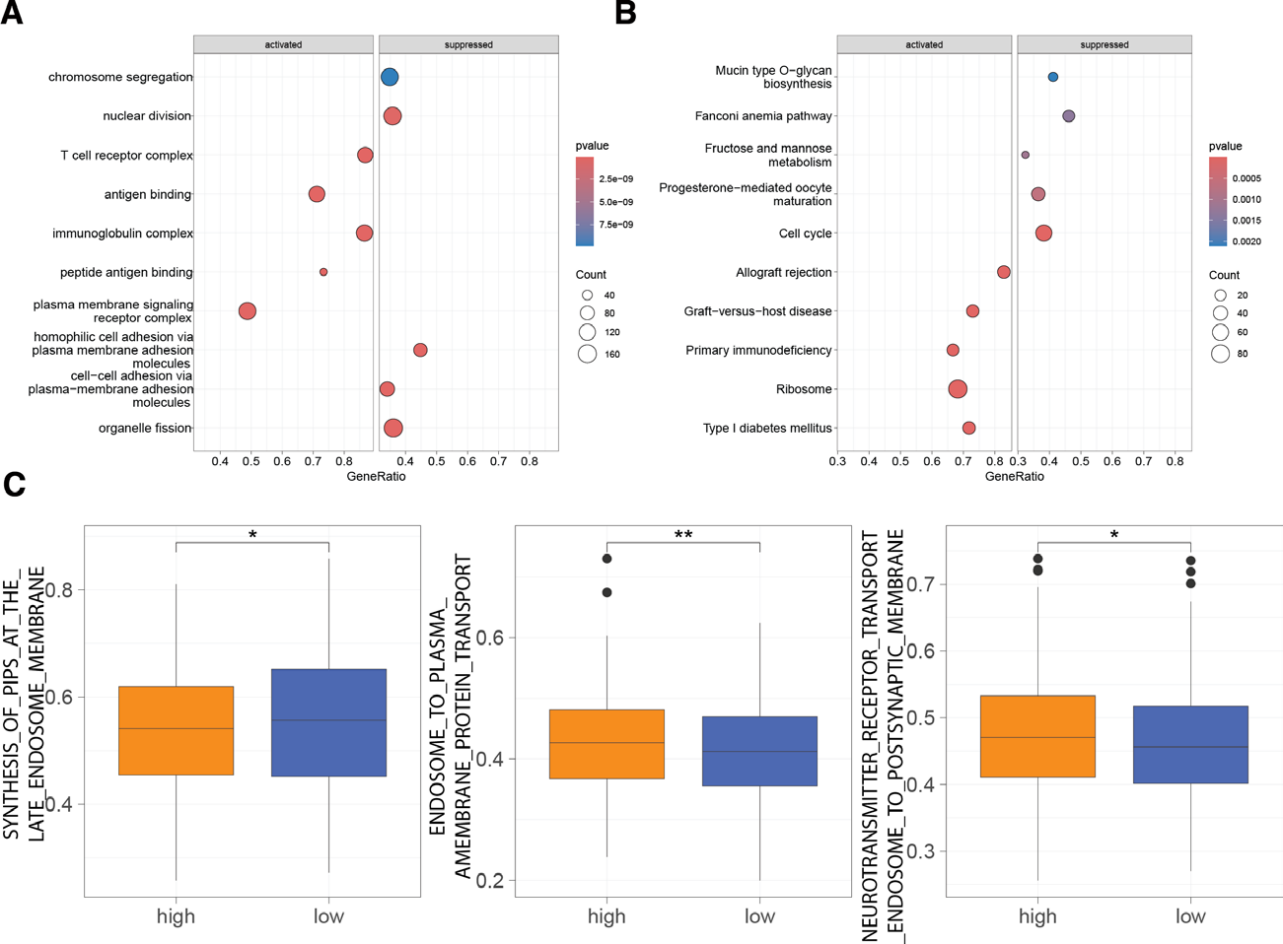
Differential gene expression profiles between high- and low-risk groups. (A) Gene Ontology (GO) enrichment analysis. (B) Kyoto Encyclopedia of Genes and Genomes (KEGG) pathway enrichment analysis. (C) Gene set variation analysis (GSVA) scores for endosome-related gene sets between high- and low-risk groups. *P < .05; **P < .01.

GSEA based on Gene Ontology gene sets showed that in comparison to the high-risk group, the low-risk group exhibited significantly increased activity in processes such as T cell receptor complex, antigen binding, immunoglobulin complex, peptide antigen binding, and plasma membrane signaling receptor complex, while organelle fission and nuclear division were suppressed (Fig. 4A). GSEA using the Kyoto Encyclopedia of Genes and Genomes gene sets revealed that ribosome activity was enhanced in the low-risk group, accompanied by dampened cell cycle pathways (Fig. 4B). Furthermore, gene set variation analysis indicated higher scores for SYNTHESIS_OF_PIPS_AT_THE_LATE_ENDOSOME_MEMBRANE and lower scores for ENDOSOME_TO_PLASMA_MEMBRANE_PROTEIN_TRANSPORT and NEUROTRANSMITTER_RECEPTOR_TRANSPORT_ENDOSOME_TO_POSTSYNAPTIC_MEMBRANE in the low-risk group compared to the high-risk group (Fig. 4C).

### 3.5. Association of the risk signature with somatic mutations

**Figure 5.**
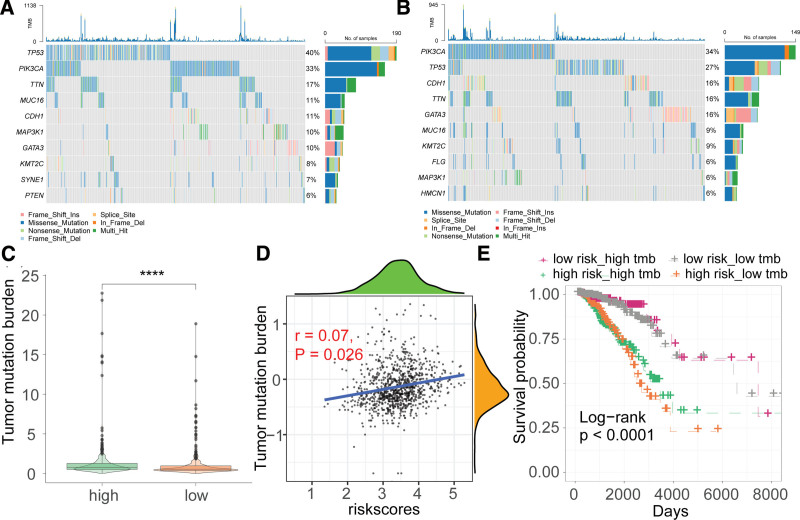
Relationship between the endosome-related genes (ERGs)-derived risk signature and somatic mutations. (A and B) Oncoplots showing somatic mutations in high- and low-risk groups. (C) Comparison of tumor mutation burden (TMB) between high- and low-risk groups. ****P < .0001. (D) Correlation analysis between risk score and TMB. (E) Kaplan–Meier (K–M) survival curves for different risk and TMB groups.

We meticulously analyzed somatic mutation data from breast cancer patients, categorizing them into high- and low-risk groups. The top 10 driver gene mutation frequencies in each group are depicted in Figure 5A and B. To delve deeper, we calculated tumor mutational burden (TMB) for each risk group based on the somatic mutation data. Strikingly, the high-risk group exhibited significantly higher TMB compared to the low-risk group (Fig. 5C). Furthermore, a positive correlation between TMB and risk score was observed (Fig. 5D). Upon stratifying TMB by its median into high TMB (high tmb) and low TMB (low tmb) groups and assessing their respective high- and low-risk prognosis, we noted significant differences in survival outcomes across these 4 groups (Fig. 5E).

### 3.6. Relationship between ERGs and treatment responsiveness

**Figure 6.**
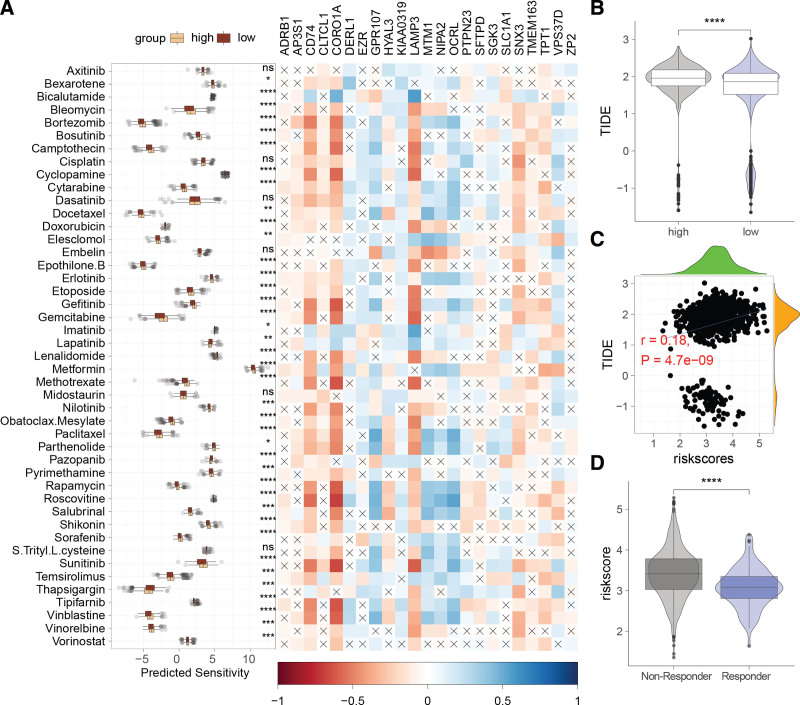
Association of the endosome-related genes (ERGs)-derived risk signature with treatment responses. (A) Relationship between the risk signature and drug sensitivity. (B) Comparison of tumor immune dysfunction and exclusion (TIDE) scores between high- and low-risk groups. (C) Scatter plot illustrating the correlation between TIDE and risk score. (D) Comparison of risk scores between nonresponders and responders to immunotherapy. ****P < .0001.

Drug sensitivity analysis (Fig. 6A) revealed that the low-risk group showed heightened sensitivity to most drugs, with the exception of thapsigargin, pazopanib, and lapatinib. Remarkably, certain ERGs, including CD74, Cofilin 1 (CORO1A), lysosome-associated membrane protein 3 (LAMP3), SNX3, TPT1, and TMEM163, were inversely correlated with drug sensitivity, whereas GPR107, MTM1, NIPA2, and PCRL displayed positive correlations. The low-risk group demonstrated a higher TIDE score (Fig. 6B), which was positively correlated with the risk score (Fig. 6C). Moreover, nonresponders to immunotherapy had significantly higher risk scores compared to responders (Fig. 6D), suggesting that patients with higher risk scores exhibit reduced responsiveness to immunotherapy.

### 3.7. Association of ERG-derived risk signature with immune landscape

**Figure 7.**
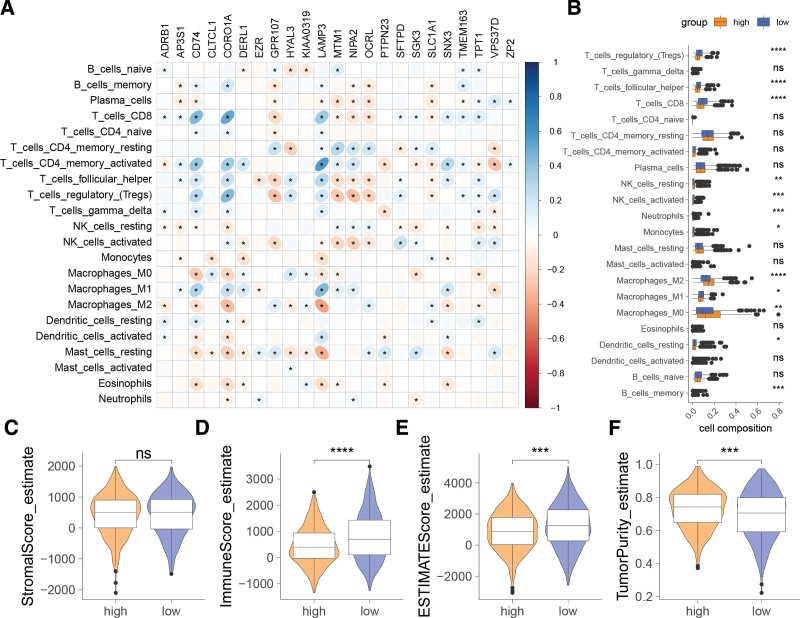
Association of the endosome-related genes (ERGs)-derived risk signature with the immune landscape. (A) Correlation of genes forming the risk signature with immune cell infiltration. (B) Differences in immune cell infiltration between high- and low-risk groups. ns = not significant; *P < .05; **P < .01; ***P < .001; ****P < .0001.

Correlation analysis illustrated significant relationships between ERGs constituting the risk signature and immune cell infiltration, with CD74, CORO1A, LAMP3, GPR107, MTM1, NIPA2, and OCRL showing correlations with various T-cell subsets. Additionally, CD74, CORO1A, and LAMP3 were associated with infiltration by macrophages, mast cells, and eosinophils (Fig. 7A). The low-risk group featured increased infiltration by T cells and M1 macrophages, along with decreased M0 and M2 macrophage populations, compared to the high-risk group (Fig. 7B). Moreover, the low-risk group had elevated immunoscores, estimate scores, and reduced tumor purity (Fig. 7C–F).

### 3.8. Construction and evaluation of the nomogram

**Figure 8.**
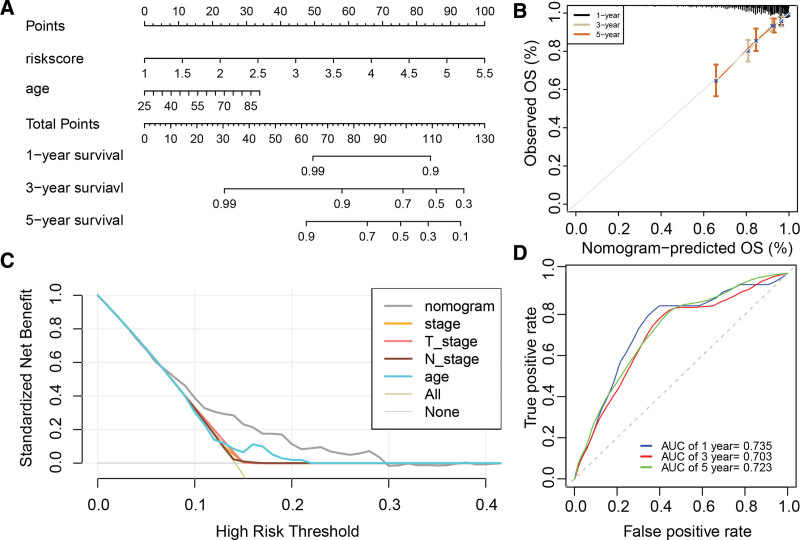
Construction and validation of a nomogram for breast cancer prognosis. (A) Nomogram integrating risk score and age for predicting 1-, 3-, and 5-year overall survival in breast cancer. (B) Calibration curves for nomogram-predicted survival probabilities. (C) Decision curve analysis comparing the net benefit of the nomogram with other predictors. (D) Receiver operating characteristic (ROC) curves evaluating the nomogram’s performance in predicting 1-, 3-, and 5-year overall survival.

Univariate Cox analysis identified the risk score [HR (95% CI): 3.4 (2.6–4.5), *P* = 4.3e−17] and age [HR (95% CI): 0.5 (0.36–0.7), *P* = 5.3e−05] as prognostic factors for breast cancer. Both were confirmed as independent prognostic indicators in the multivariate Cox analysis (Table 1). Consequently, we constructed a nomogram incorporating the risk score and age to predict 1-, 3-, and 5-year overall survival in breast cancer (Fig. 8A). Calibration curves demonstrated a good fit between predicted and actual survival probabilities (Fig. 8B). The nomogram showed a higher standardized net benefit compared to other factors in prognostic assessment (Fig. 8C) and achieved AUC values of 0.735, 0.703, and 0.723 for predicting 1-, 3-, and 5-year survival, respectively (Fig. 8D), highlighting its robust performance in breast cancer prognosis.

**Table 1:** Univariate and multivariate Cox regression analysis for prognostic relevance of risk score and clinicopathological features.

Characteristics	Univariate Cox		Multivariate Cox	
	HR [95% CI]	*P*-value	HR [95% CI]	*P*
Risk score	3.4 [2.6–4.5]	4.3e−17	3.25 [2.43, 4.33]	<.000001
Age	0.5 [0.36–0.7]	5.3e−05	0.59 [0.42, 0.83]	.002117
Gender	1.7 [0.23–12]	0.62	1.55 [0.21, 11.38]	.66671
Stage	1.1 [0.9–1.4]	0.28	1.03 [0.63, 1.68]	.902352
T_stage	0.99 [0.79–1.2]	0.94	0.90 [0.65, 1.25]	.543151
N_stage	1.1 [0.93–1.4]	0.21	1.07 [0.79, 1.45]	.651449

### 3.9. Validation of ERG expression

**Figure 9.**
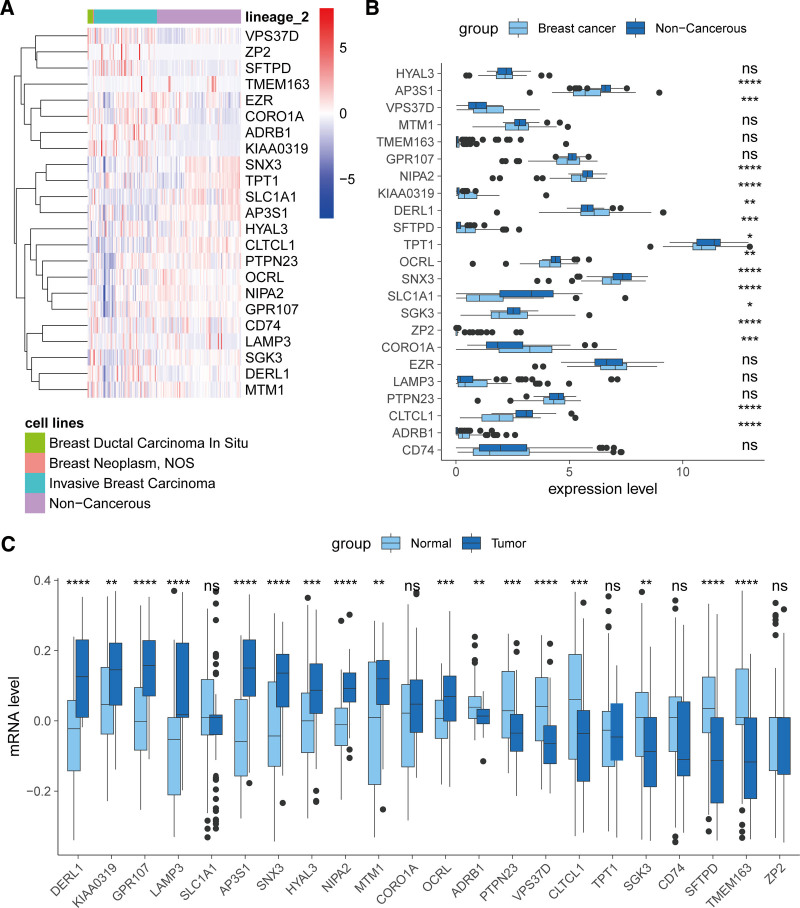
Validation of risk score-associated endosome-related genes (ERGs). (A) Heatmap illustrating the mRNA level of ERGs in breast cancer and noncancerous cell lines. (B) Comparison of the expression of ERGs between breast cancer and noncancerous cell lines. (C) Comparison of the expression of ERGs between breast cancer and adjacent normal tissues in the GSE14999 cohort. ns = not significant, *P < .05; **P < .01; ***P < .001; and ****P < .0001.

To validate the expression patterns of risk score-associated ERGs, we conducted a comparative analysis of gene expression data from breast cancer cell lines and nonmalignant cell lines obtained from the Cancer Cell Line Encyclopedia. Our results identified 15 ERGs that were significantly differentially expressed in breast cancer cell lines. Among these, the expression of AP3S1, NIPA2, TPT1, OCRL, SNX3, SLC1A1, SGK3, and Clathrin heavy chain-like 1 (CLTCL1) was markedly decreased, while VPS37D, KIAA0319, DERL1, SFTPD, CORO1A, ZP2, and adrenoceptor beta 1 exhibited increased expression levels. Importantly, we observed considerable heterogeneity in the expression of these genes across distinct breast cancer cell lines, underscoring the complexity of the disease (Fig.9A and B). To extend our findings to a clinical context, we further analyzed the expression of these ERGs in the GSE14999 breast cancer cohort. In this cohort, 18 ERGs were found to be aberrantly expressed in breast cancer tissues, with 10 genes being significantly upregulated and 8 genes significantly downregulated. Notably, the expression trends of KIAA0319, DERL1, SGK3, and CLTCL1 in tumor tissues mirrored those observed in the cell lines, providing consistency between in vitro and in vivo observations (Fig. 9C). These findings highlight the potential of these ERGs as biomarkers for breast cancer prognosis and suggest their involvement in the molecular mechanisms underlying the disease. Further investigation into the functional roles of these genes may provide insights into therapeutic targets and personalized treatment strategies.

## 4. Discussion

Multigene prognostic assessment models provide a robust tool for tailoring individualized treatment strategies for breast cancer patients,^[19–21]^ yet an ERG-based risk profile has not been established. In this study, the transcriptomic and clinical data from TCGA and University of California Santa Cruz database databases was used to identify molecular signatures with clinical relevance. Through Lasso Cox regression, this study identified a subset of ERGs that effectively stratify patients into high- and low-risk groups. This risk signature, validated across independent cohorts, highlights the robustness of the identified ERGs in predicting survival outcomes, emphasizing the importance of considering endosomal biology in breast cancer prognosis. Relative to alternative polygenic risk profiles, our predictive model exhibits markedly enhanced performance in assessing patient outcomes.^[22–25]^ Therefore, the integration of our model with prognostic features identified through the Surveillance, Epidemiology, and End Results (SEER) program and clinical cohort studies is poised to significantly refine and improve the prognostic evaluation for patients with breast cancer.^[26,27]^

In our study, we have identified a multigenic prognostic signature comprising 23 genes that play pivotal roles in the progression and prognosis of breast cancer. Adrenoceptor beta 1, previously reported to be implicated in the progression of various cancers, undergoes expression changes that can impact tumor growth, angiogenesis, and responsiveness to therapy.^[28,29]^ CD74, apart from its role in the immune system,^[30]^ is involved in multiple signaling pathways and promotes cell proliferation and migration in certain cancers.^[31]^ In the context of breast cancer, aberrant expression of CD74 may be associated with disease progression and poor prognosis.^[32]^ CLTCL1, encoding a protein implicated in cell cycle regulation and chromosome stability, may facilitate tumorigenesis upon overexpression.^[33]^ TPT1, by activating the AKT/mTOR signaling pathway and enhancing protein synthesis, fosters the proliferation and survival of cancer cells.^[34]^

The integration of immune infiltration analysis via CIBERSORT and ESTIMATE algorithms adds a new dimension to our understanding of the TME in relation to ERG expression. The observation that low-risk patients exhibit increased infiltration of T cells and M1 macrophages, alongside suppression of cell cycle pathways and activation of antigen presentation machinery, suggests a more favorable immune contexture. These findings align with the emerging concept that an immunologically “hot” TME, characterized by T cell infiltration and immune activation, is associated with better clinical outcomes.^[35]^ In the risk profiles, CD74 not only partakes in the canonical antigen presentation process but also significantly contributes to modulating immune cell functions. Within the tumor microenvironment, the expression of CD74 is often upregulated, influencing the activation and function of immune cells, particularly T cells, which at times facilitates immune evasion.^[30]^ Given its pivotal role in tumor immunoregulation, CD74 has emerged as a focal point for the development of immunotherapeutic strategies. For instance, the presence of HLA-DR + CD74 + neutrophils has been correlated with favorable prognoses in cancer patients; these cells are capable of inducing antigen-specific T-cell responses and fostering a “hot tumor” microenvironment conducive to immunotherapy, characterized by enhanced immune activation.^[36,37]^ Therefore, the ERG signature could serve as a valuable tool for predicting immune responsiveness and guiding immunotherapy strategies.

Drug sensitivity analyses, as demonstrated by the varying responses to multiple chemotherapeutic agents, underscore the potential of the ERG signature to predict treatment efficacy. Notably, the differential sensitivity to drugs like thapsigargin, pazopanib, and lapatinib in high- and low-risk groups indicates that the ERG profile might inform drug selection, thereby personalizing treatment regimens. The association of specific ERGs with drug sensitivity profiles, such as negative correlations with CD74, CORO1A, LAMP3, and positive correlations with GPR107, MTM1, and NIPA2, opens avenues for exploring the mechanistic basis of these interactions and developing targeted interventions. It has been demonstrated that CD74 is implicated in regulating drug resistance across various cancers, including non-small cell lung cancer^[38]^and gliomas,^[39]^ where its genetic fusion with ROS1 is prevalent. This fusion leads to aberrant activation of ROS1 tyrosine kinase, rendering patients highly susceptible to ROS1 inhibitors.^[40]^ LAMP3 is involved in the autophagic process and has been shown to contribute to tamoxifen resistance in breast cancer. Studies have revealed that knockdown of LAMP3 in tamoxifen-resistant cells restores sensitivity to tamoxifen.^[41]^ CORO1A, expressed in T cells, plays a crucial role in T cell activation, migration, and the formation of the immunological synapse. Evidence suggests that CORO1A may influence the efficiency of T cell immune responses against tumors by modulating signaling pathways and cytoskeletal dynamics within these cells.^[42,43]^

The nomogram integrating the risk score with age presents a clinically practical tool for survival prediction. Its performance, as evidenced by the calibration curves, standardized net benefit analysis, and AUC values, underscores the clinical utility of this risk stratification approach. By incorporating easily accessible patient information, the nomogram enables clinicians to provide more accurate prognostic assessments and guide treatment decisions.

While the study has made significant strides, several areas warrant further exploration. Elucidating the functional roles of the identified ERGs in breast cancer pathogenesis and their mechanistic involvement in modulating immune response and drug sensitivity is crucial. Investigating the interplay between ERGs and other molecular pathways, such as endoplasmic reticulum stress response, autophagy, and epigenetic modifications, may reveal additional layers of complexity in breast cancer biology. Additionally, the impact of the ERG signature on response to immunotherapy, especially checkpoint inhibitors, needs to be systematically evaluated given the observed correlations with immune infiltration and TIDE scores.

## 5. Conclusion

In summary, this study comprehensively elucidates the expression, mutation patterns, and prognostic characteristics of ERGs in breast cancer. We have successfully constructed and validated a risk signature comprised of 23 ERGs, which holds promise for predicting patient outcomes and informing therapeutic responses in breast cancer. Integrating age as a clinical feature, we developed a clinically applicable nomogram. However, further biological experiments and clinical investigations are warranted to deepen our understanding of the underlying biological functions of ERGs and to ascertain the robustness of the identified risk signature.

## Author contributions

**Conceptualization:** Guowei Jiang, Ye Wang.

**Data curation:** Guowei Jiang, Ye Wang.

**Formal analysis:** Guowei Jiang, Ye Wang.

**Funding acquisition:** Guowei Jiang, Ye Wang.

**Investigation:** Guowei Jiang, Ye Wang.

**Methodology:** Guowei Jiang, Ye Wang.

**Project administration:** Guowei Jiang, Ye Wang.

**Resources:** Guowei Jiang, Ye Wang.

**Software:** Guowei Jiang, Ye Wang.

**Supervision:** Guowei Jiang, Ye Wang.

**Validation:** Guowei Jiang, Ye Wang.

**Visualization:** Guowei Jiang, Ye Wang.

**Writing – original draft:** Guowei Jiang, Ye Wang.

**Writing – review & editing:** Guowei Jiang, Ye Wang.

## Supplementary Material


